# Role of lncRNA BANCR in Human Cancers: An Updated Review

**DOI:** 10.3389/fcell.2021.689992

**Published:** 2021-08-02

**Authors:** Bashdar Mahmud Hussen, Tahereh Azimi, Atefe Abak, Hazha Jamal Hidayat, Mohammad Taheri, Soudeh Ghafouri-Fard

**Affiliations:** ^1^Department of Pharmacognosy, College of Pharmacy, Hawler Medical University, Erbil, Iraq; ^2^Phytochemistry Research Center, Shahid Beheshti University of Medical Sciences, Tehran, Iran; ^3^Men’s Health and Reproductive Health Research Center, Shahid Beheshti University of Medical Sciences, Tehran, Iran; ^4^Department of Biology, College of Education, Salahadddin University-Erbil, Erbil, Iraq; ^5^Skull Base Research Center, Loghman Hakim Hospital, Shahid Beheshti University of Medical Sciences, Tehran, Iran; ^6^Department of Medical Genetics, Shahid Beheshti University of Medical Sciences, Tehran, Iran

**Keywords:** BANCR, lncRNA, biomarker, cancer, expression

## Abstract

Being located in a gene desert region on 9q21.11-q21.12, BRAF-activated non-protein coding RNA (BANCR) is an lncRNA with 693 bp length. It has been discovered in 2012 in a research aimed at assessment of gene expression in the melanocytes in association with *BRAF* mutation. Increasing numbers of studies have determined its importance in the tumorigenesis through affecting cell proliferation, migration, invasion, apoptosis, and epithelial to mesenchymal transition. BANCR exerts its effects via modulating some tumor-related signaling pathways particularly MAPK and other regulatory mechanisms such as sponging miRNAs. BANCR has been up-regulated in endometrial, gastric, breast, melanoma, and retinoblastoma. Conversely, it has been down-regulated in some other cancers such as those originated from lung, bladder, and renal tissues. In some cancer types such as colorectal cancer, hepatocellular carcinoma and papillary thyroid carcinoma, there is no agreement about BANCR expression, necessitating the importance of additional functional studies in these tissues. In the present manuscript, we review the investigations related to BANCR expression changes in cancerous cell lines, clinical samples, and animal models of cancer. We also discuss the outcome of its deregulation in cancer progression, prognosis, and the underlying mechanisms of these observations.

## Introduction

Long non-coding RNAs (lncRNAs) which are RNA molecules sized more than 200 nucleotides, play significant roles in controling gene expression through epigenetic, transcription, and post-transcription modifications. Due to their regulatory functions, they can be important as effective factors in diseases pathogenesis including cancers (Jiang et al., 2019). BRAF-activated non-protein coding RNA (BANCR, Gene ID: 100885775) is an lncRNA with 693 bp length (Flockhart et al., 2012), that is composed of 4 exon and is located on 9q21.11-q21.12, a gene desert region^1^. It has been discovered in 2012 as a result of Flockhart et al. (2012) research on melanocytes with and without *BRAF* mutation. The observed up-regulation of BANCR in melanoma, has been a starting point for subsequent expression studies in diverse types of cancers. Increasing numbers of studies about BANCR in various cancers have determined its importance in the tumorigenesis through affecting cell proliferation, migration, invasion, apoptosis, and epithelial to mesenchymal transition (EMT), which are crucial for disease prognosis. BANCR exerts its effects via modulating some tumor-related signaling pathways particularly MAPK and other regulatory mechanisms such as sponging microRNAs (miRNAs).

The role of BANCR in cancer has been previously reviewed (Yu et al., 2017; Zou et al., 2017). In the present manuscript, we provide an update on investigations related to BANCR expression changes in cancerous cell lines, clinical samples and animal models of cancer. We also discuss the outcome of its deregulation in cancer progression, prognosis and the underlying mechanisms of these observations.

## *In vitro* Studies

**FIGURE 1:**
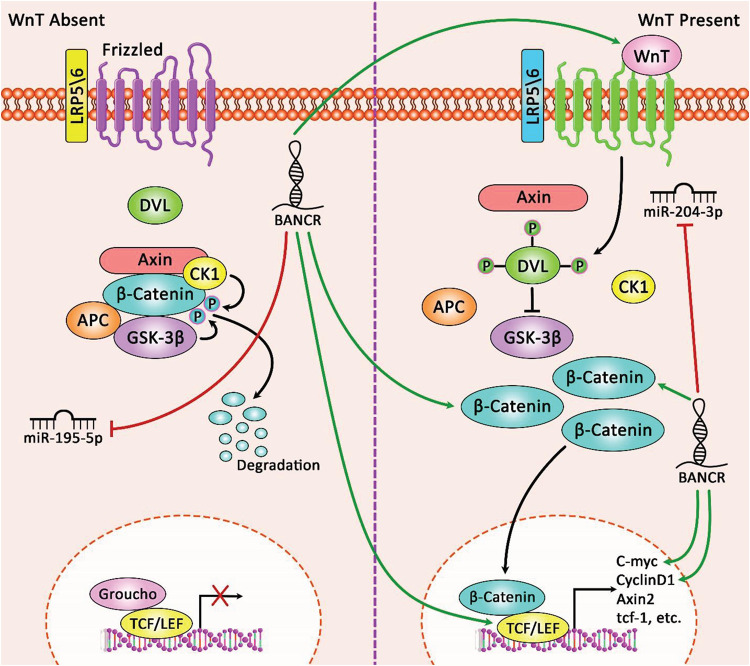
A schematic diagram of the oncogenic role of lncRNA BANCR in regulating Wnt/β-catenin signaling pathway in pancreatic cancer and retinoblastoma. Accumulating research has figured out that lncRNA BANCR could play a crucial role in triggering the activation of Wnt/β-catenin cascade in several tumor cells through sponging various miRNAs. As an illustration, this lncRNA could elevate pancreatic cancer tumorigenesis through up-regulating expression levels of β-catenin, c-Myc, and cyclinD1 by miR-195-5p/Wnt/β-catenin axis in target cells (Wu et al., 2019). In addition, overexpression of lncRNA BANCR through regulating Wnt/β-catenin pathway could promote cell proliferation, apoptosis, invasion, and migration in retinoblastoma. In fact, this lncRNA via sponging miR-204-3p could have an effective part in enhancing the expression levels of Wnt4, β-catenin, and TCF4 in tumor cells (Sun et al., 2020). Green arrows indicate the up-regulation of target genes modulated via lncRNA BANCR and red arrows depict inhibition of miRNAs regulated by this lncRNA.

BRAF-activated non-protein coding RNA silencing has remarkably repressed proliferation, migration, and invasion of endometrial cancer. These functional effects have been accompanied by inhibition of ERK/MAPK signaling pathway and down-regulation of MMP2 and MMP1 levels (Wang D. et al., 2016). An experiment in the uterine leiomyoma has indicated the effect of anti-cancer agent deoxyelephantopin in down-regulation of a number of oncogenic lncRNAs including BANCR (Pandey et al., 2020). Another study has reported over-expression of BANCR in colorectal cancer cells and verified the impact of ectopic expression of BANCR in enhancement of migratory potential of human CRC Caco-2 cells. Besides, BANCR silencing has suppressed the migratory aptitude of the HCT116 cells. The effects of BANCR on cancer cell migration are mediated through induction of EMT via an MEK/ERK-dependent route (Guo et al., 2014). Another functional study has demonstrated up-regulation of BANCR in gastric cancer cells. BANCR silencing has blocked gastric cancer growth and boosted apoptosis possibly through decreasing NF-κB1 (P50/105) levels and activity. miR-9 has been shown to target NF-κB1, therefore anta-miR-9 has upturned the impact of BANCR on gastric cancer cell proliferation and death (Zhang et al., 2015). BANCR has also been shown to be up-regulated in a hepatocellular cancer cell line compared with normal hepatic cells. BANCR silencing has substantially repressed proliferation and colony construction capability, arrested cells, and induced apoptosis in hepatocellular cancer cells. BANCR silencing has also diminished the activity of MEK, ERK and JAK signaling pathways (Li et al., 2017). Another studies in liver cancer cells has demonstrated the impact of BANCR silencing in impairment of cell proliferation, promotion of cell apoptosis, reduction of invasive properties and down-regulation of vimentin, and up-regulation of E-cadherin expression (Zhou and Gao, 2016). Functional studies in pancreatic cancer cells have not only confirmed the oncogenic effects of BANCR, but also has identified miR-195-5p as a direct target of BANCR through which this lncRNA regulates Wnt/β-catenin pathway (Wu et al., 2019). Figure 1 depicts the role of lncRNA BANCR in modulating the Wnt/β-catenin signaling pathway and its impact on tumorigenesis of cancer cells via sponging miR-195-5p and miR-204-3p.

Similarly, BANCR silencing in MCF-7 cells has suppressed cell proliferation and colony construction ability, while promoting cell apoptosis which has been evident through the observed up-regulation of BAX, cleaved-Caspase-3 and PARP. Moreover, BANCR silencing has affected invasive and metastatic capabilities of MCF-7 cells through suppression of EMT and reduction of MMP levels (Lou et al., 2018).

**FIGURE 2:**
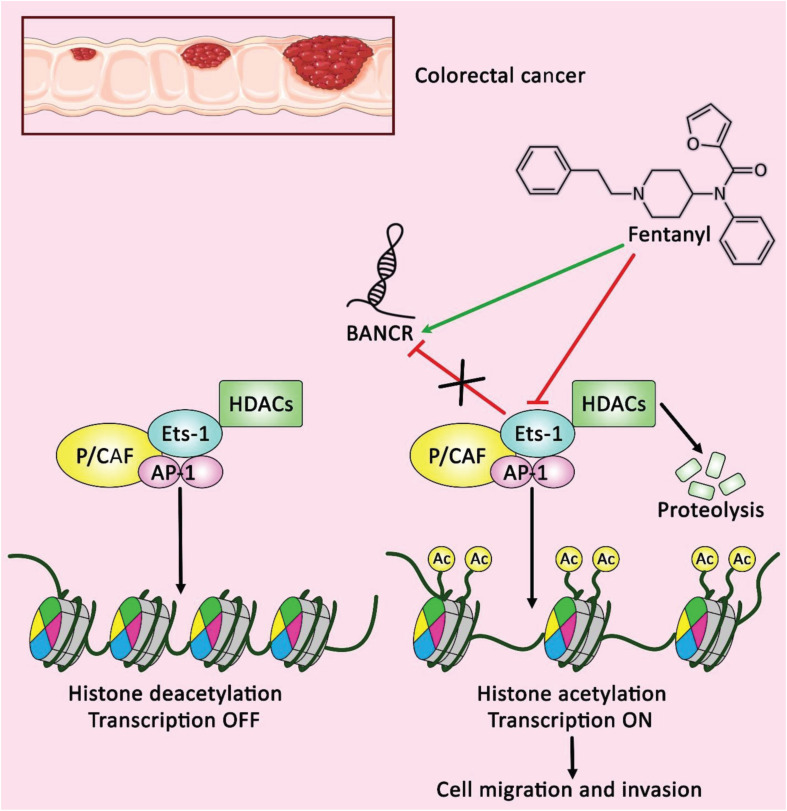
A schematic representation of the potential role of fentanyl as an anti-tumor drug in colorectal cancer cells. Accumulating evidence illustrates that Ets-1 could negatively regulate the expression of BANCR through the deacetylation of histones H3 within BANCR promoter. The regulatory role of fentanyl on reducing the expression level of Ets-1 and inducing BANCR upregulation could have a significant part in decreasing cell clonal formation, as well as cell migration and invasion in colorectal cancer (Li A.-x. et al., 2015). Therefore, lncRNA BANCR could play an effective role as a tumor suppressor in the regulation of colorectal cancer cells (Li A.-x. et al., 2015). Green arrow indicates the regulatory role of fentanyl on the overexpression of lncRNA BANCR, red arrows depict the inhibitory role of fentanyl on the expression of Ets-1, and the impact of Ets-1 on down-regulating the expression level of BANCR.

On the other hand, BANCR has tumor suppressor effects in a number of cancers. In bladder cancer cell lines, over-expression of BANCR has repressed cell proliferation, stimulated apoptotic pathways and suppressed migration (He et al., 2016). Similarly, expression of BANCR has been decreased in renal cancer cells compared with normal human proximal tubule epithelial cells. Ectopic up-regulation of BANCR has led to repression of proliferation, migration and invasiveness these cells, while increasing apoptosis and arresting cells at G1 (Xue et al., 2018). Up-regulation of BANCR has also inhibited colorectal cancer cell proliferation, activated G0/G1 arrest and enhanced apoptosis via influencing expression of p21 at posttranscriptional level (Shi et al., 2015). Another study in colorectal cancer cells has revealed the effects of Fentanyl on induction of BANCR over-expression and Ets-1 under-expression (Figure 2). Notably, Ets-1 has been shown to decrease BANCR expression through deacetylation of histones H3 inside the promoter region of this lncRNA. Fentanyl has decreased colony establishment and suppressed cell migration and invasion in colorectal cancer cells, whereas Ets-1 up-regulation has suppressed fentanyl-associated influences through down-regulation of BANCR (Li A.-x. et al., 2015). Supplementary Table 1 shows the abnormal levels of BANCR in cancer cell lines and the functional consequences of this dysregulation.

## Animal Studies

**FIGURE 3:**
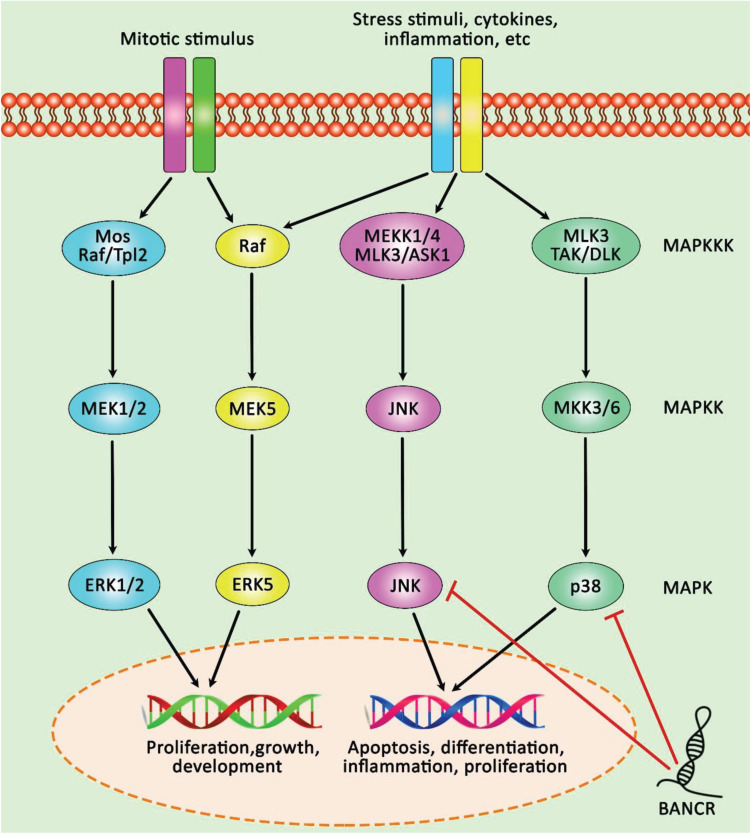
A schematic illustration of tumor suppressive role of lncRNA BANCR in inhibiting proliferation and migration of lung carcinoma through MAPK pathways. LncRNA BANCR via suppressing the activation of p38 MAPK and JNK and modulating MAPK cascades could effectively reduce cell proliferation and migration of lung carcinoma (Jiang et al., 2015). Red arrows illustrate downregulation of target genes modulated via lncRNA BANCR.

The consequences of BANCR over-expression and down-regulation on tumor growth have been appraised *in vivo*. For instance, Ma et al. (2018) have produced xenograft model of colorectal cancer through injection of LoVo cells into animals to appraise the impact of BANCR silencing on tumor growth and response to Adriamycin. They have shown that administration of Adriamycin or BANCR silencing can decrease tumor growth. Besides, BANCR silencing has increased anti-cancer effects of this agent in animal models. The effects of BANCR has been shown to be mediated through miR-203/CSE1L axis (Ma et al., 2018). However, these results are not in agreement with the results of Shi et al. (2015) study. These authors have injected BANCR-overexpressing HCT116 cells into the nude mice. They have reported significant smaller size of tumors in this group of animals compared with those injected with HCT116 cells transfected with empty vector. Histopathological examination of tumor tissues of the former group of animals has shown karyopyknosis and shape change in the tumor samples and lower proliferation index Ki67 levels, indicating the impact of BANCR overexpression on decreasing tumor growth *in vivo* (Shi et al., 2015). Such inconsistencies also exist in other types of cancers. For instance, while a study in the xenograft model of thyroid cancer has shown the impact of BANCR up-regulation on enhancement of tumor growth (Wang et al., 2018b), another study has reported the opposite results (Liao et al., 2017). However, four independent studies in lung cancer have verified the role of BANCR silencing in suppression of tumor growth or metastatic ability, demonstrating a tumor suppressor role for this lncRNA in this type of cancer (Sun et al., 2014; Chen et al., 2015; Jiang et al., 2015; Yang and Liu, 2019). Figure 3 depicts the involving of BANCR in the regulation of MAPK pathways via p38 MAPK and JNK inactivations in lung carcinoma. Table 1 shows the consequences of BANCR up-/down-regulation in animal models of cancer.

**TABLE 1:** Consequences of BANCR up-/down-regulation in animal models of cancer.

Cancer	Animal model	Effects	References
Colorectal cancer	BALB/c male mice	Δ BANCR: ↓ tumor growth and ↑ Adriamycin sensitivity	Ma et al., 2018
	BALB/c male nude mice	↑ BANCR: ↓ tumor growth	Shi et al., 2015
Gastric cancer	Nude mice	Δ BANCR: ↓ tumor growth and volume	Zhang et al., 2015
	Female nude mice	Δ BANCR: ↓ tumor sizes, mass and ↑ sensitivity to cisplatin therapy.	Miao et al., 2021
Lung carcinoma	BALB/c male nude mice	↑ BANCR: ↓ tumor mass weight	Jiang et al., 2015
	C57B6/L male mice	Radiation therapy functions through BANCR up-regulation to inhibit tumor growth.	Chen et al., 2015
Non-small cell lung cancer	Athymic male mice	↑ BANCR: ↓ number of metastatic nodules	Sun et al., 2014
	BALB/c male nude mice	↑ BANCR: ↓ tumor growth and weight	Yang and Liu, 2019
Papillary thyroid carcinoma	Specific pathogenic free (SPF) female BALB/c nude mice	↑ BANCR: ↓ tumor growth	Wang et al., 2018b
	Specific pathogen-free (SPF) female athymic nude mice	↑ BANCR: ↓ tumor weight and size	Liao et al., 2017
	Athymic nude mice	Luteolin treatment caused BANCR down-regulation and had anti-tumor effect	Liu et al., 2017
Oral squamous cell carcinoma	Male mice	Δ BANCR: ↓ cell proliferation and tumor volume	Yao et al., 2019
Retinoblastoma	BALA/C athymic nude mice	Δ BANCR: ↓ tumorigenesis	Sun et al., 2019
Melanoma	nude mice	Δ BANCR: ↓ tumor growth	Cai et al., 2017
	BALB/c male nude mice	Δ BANCR: ↓ tumor growth and weight	Li et al., 2014

## Clinical Studies

**FIGURE 4:**
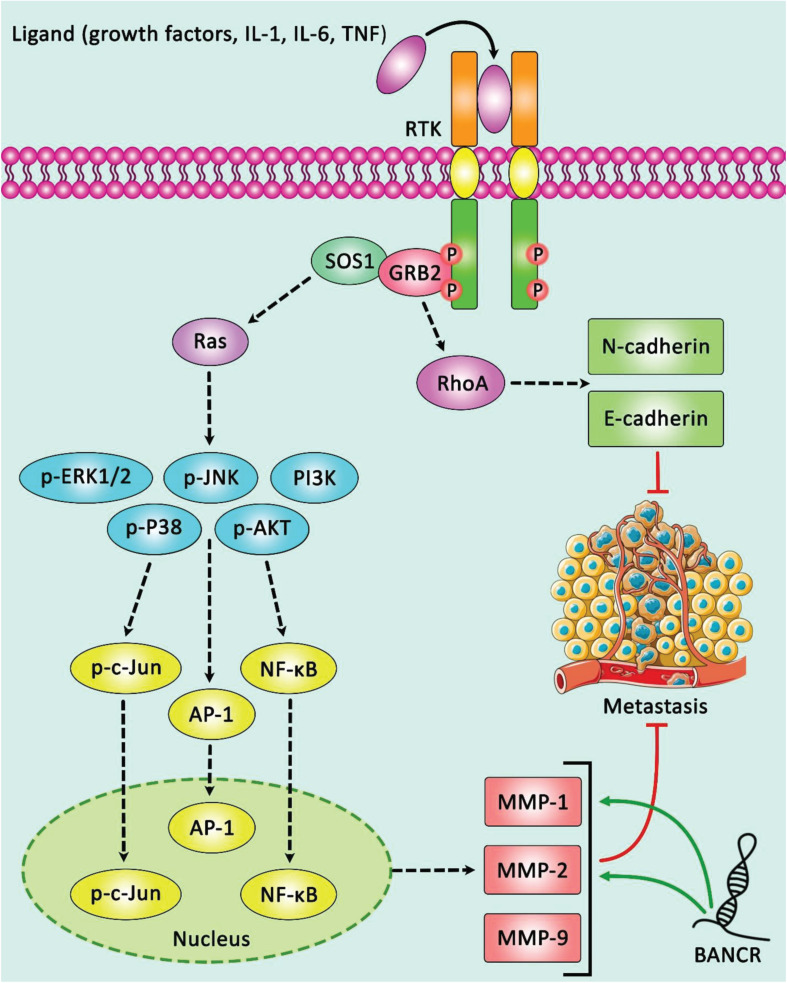
A schematic diagram of oncogenic role of lncRNA BANCR in modulating the expression of MMP2 and MMP1 via ERK/MAPK cascade in endometrial cancer. LncRNA BANCR could up-regulate in type 1 endometrial cancer tissues, and play an effective role in the progression of this type of tumor. This lncRNA via activating ERK/MAPK signaling pathway could promote the expression levels of MMP2 and MMP1, and thereby suppressing cell proliferation, migration, and invasion of EC cells (Wang D. et al., 2016). Green arrows indicate the up-regulation of target genes modulated via lncRNA BANCR.

Expression of BANCR has been elevated in type 1 endometrial cancer in correlation with FIGO stage, pathological grade, myometrial invasion, lymph node involvement, and expression levels of MMP2 and MMP1 (Figure 4; Wang D. et al., 2016). Besides, BANCR has been commonly up-regulated in colorectal cancer samples in association with lymph node involvement and clinical stage (Guo et al., 2014). High throughput lncRNA profiling in plasma samples obtained from patients with gastric cancer has led to identification of an lncRNA-based diagnostic panel consisting BANCR and a number of other lncRNAs. Notably, the diagnostic power of this panel has been superior to the CEA-based panel. Additionally, expression of these lncRNAs has been reduced by postoperative day 14, representing its aptitude to observe tumor dynamics (Zhang et al., 2017). Over-expression of BANCR in hepatocellular carcinoma cells has been correlated with advanced grade, large tumor dimension, venous intrusion, advanced clinical stage, and poor overall survival (Zhou and Gao, 2016). BANCR expression has also been elevated in breast cancer samples compared with para-carcinoma sections. Low-expressing BANCR group has better survival compared with the other patients (Lou et al., 2018).

In contrast, BANCR expression has been displayed to be down-regulated in bladder cancer tissues compared with neighboring non-tumoral sections. Among all assessed clinical and pathological features, down-regulation of BANCR has been correlated with TNM stage (He et al., 2016). Similarly, BANCR levels have been shown to be reduced in renal cell carcinoma samples compared with neighboring unaffected kidney samples in correlation with poor prognosis implying the appropriateness of BANCR as a new prognostic marker in renal cell carcinoma (Xue et al., 2018). Moreover, BANCR has been shown to be diminished in colorectal cancer tissues compared with unaffected sample. Notably, tumors with larger dimensions, which signify their higher burden, or more advanced stages have been shown to have more prominent reduced levels of BANCR indicating the importance of BANCR as a suitable diagnostic marker for early stages of this type of cancer (Shi et al., 2015). Contrary to the study conducted by Zhou and Gao (2016) and Zhao et al. (2018) have demonstrated down-regulation of BANCR in hepatitis B virus-associated liver cancer in association with α-fetoprotein concentration and number of tumors.

Another study has revealed association between the presence of *BRAF* V600E mutation and with lower levels of BANCR in papillary thyroid carcinoma. Compared with the corresponding non-cancerous tissues, relative expression of BANCR has been lower in *BRAF* mutation positive samples, while it has been unchanged or elevated in *BRAF* mutation negative samples. In samples harboring *BRAF* V600E mutation, up-regulation of BANCR has been correlated with lymph node involvement, while in *BRAF* V600E negative samples, over-expression of this lncRNA has been linked with invasion of tumor to thyroid capsule. Thus, BANCR has been suggested as a valuable prognostic marker in risk stratification in papillary thyroid cancer (Stojanović et al., 2020). Moreover, high BANCR expression in papillary thyroid cancer has been associated with higher probability of extracapsular invasion and lateral LNM in tumors and a lower probability of bilateral tumors and multifocality in the nearby non-cancerous tissues. Contrary to the formerly mentioned study, the presence of *BRAF* V600E mutation has been associated with higher probability of BANCR up-regulation. Patients with *BRAF* V600E mutation, over-expression of BANCR and down-regulation of miR-9 have been shown to need earlier surgical management particularly total thyroidectomy in primary surgery in order to decrease tumor recurrence (Shi et al., 2020). Supplementary Table 2 demonstrates the observed effects of BANCR dysregulation in clinical settings.

A previous meta-analysis of 11 published papers on 1240 cancer samples has shown lower overall survival of patients who had BANCR over-expression. However, this study did not show association between expression of BANCR and relapse-free, disease-free or progression-free survival times. Besides BANCR over-expression has been found to be correlated with lymph node involvement and remote metastasis (Zhang and Cai, 2019).

## Discussion

BRAF-activated non-protein coding RNA is an lncRNA that plays regulatory roles in the tumorigenesis. According to the mentioned studies, it plays a dual role in the carcinogenesis. BANCR has been up-regulated in numerous types of cancers such as endometrial, gastric, breast, melanoma, and retinoblastoma. Conversely, it has been down-regulated in some other cancers such as those originated from lung, bladder and renal tissues, and has been shown to exert negative effects on tumorigenesis in these tissues. Therefore, BANCR may function as an oncogene or tumor-suppressor gene in a context-dependent manner. This mode of function might be explained by its effects of tissue-specific miRNAs or transcription factors. In other words, expression levels of target miRNAs or transcription factors in each tissue might define the oncogenic or tumor suppressor role of BANCR. Tumor suppressor role of BANCR is mediated through modulation of p38 MAPK, p21, ERK, PI3K-Akt, and Wnt pathways. In some cancer types such as colorectal cancer, hepatocellular carcinoma and papillary thyroid carcinoma, there is no agreement about BANCR expression, necessitating the importance of additional functional studies in these tissues.

**FIGURE 5:**
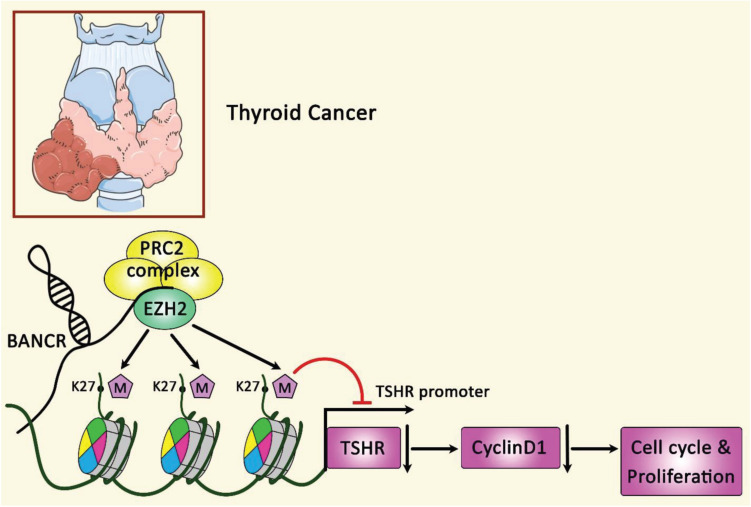
A schematic summary of the role of BANCR and EZH2 modulation on the expression of cyclin D1. LncRNA BANCR could regulate growth and cell cycle of papillary thyroid carcinoma via the modulation of cyclin D1 protein expression. Furthermore, via physical binding of BANCR to EZH2, this lncRNA could regulate TSHR and the following target genes especially cyclin D1 (Zheng et al., 2016). BANCR has an oncogenic role in the modulation of PTC cancer (Zheng et al., 2016).

Figure 5 illustrates the regulation of cyclin D1 through BANCR and EZH2 in papillary thyroid carcinoma.

Investigations in clinical samples have demonstrated significant association between BANCR expression and overall, disease-free, and relapse-free survival rates. Moreover, these studies have represented its importance as a prognostic factor. *In vivo* studies in animal models have also confirmed the role of BANCR deregulation in tumor progression.

BRAF-activated non-protein coding RNA serves as a sponge for miR-203, miR-9, miR-195-5p, miR-338-3p, miR-204-3p, and miR-204. These interactions enable BANCR to affect crucial signaling pathways in the processes of carcinogenesis namely MAPK, PI3K/AKT, NF-κB, and WNT/β-catenin pathways. The tissues-specific effects of BANCR might be explained by the relative abundance of these miRNAs in different tissues.

Animal studies have shown the impact of modulation of BANCR expression on tumor growth. Moreover, this lncRNA can influence the response of malignant cells to the chemotherapeutic agent Adriamycin. Yet, these studies have not been replicated in clinical settings possibly due to inconsistent results of animal studies. On the other hand, BANCR has been shown to be involved in the therapeutic effects of some anti-cancer substances such as Fentanyl and deoxyelephantopin signifying the importance of identification of the possible involved mechanisms to apply these substances in the clinical settings. Besides, BANR has been shown to be implicated in the response of lung cancer cells to radiotherapy (Chen et al., 2015). Notably, expression levels of BANCR can be used for establishment of personalized methods of cancer treatment and stratification of patients based on the risk of tumor recurrence. This field has been explored in papillary thyroid cancer, yet other types of cancers lack informative data in this regard.

The observed associations between expression levels of BANCR and *BRAF* mutation in the melanoma and papillary thyroid cancer implies the regulatory role of this gene expression of BANCR. However, the association between *BRAF* mutations and expression of this lncRNA has been less studied in other malignancies. Meanwhile, the transcription factor Ets-1 has been shown to regulate BANCR expression through induction of histone deacetylation. Other regulatory mechanisms for BANCR expression should be clarified in future studies. Comparison of epigenetic marks in BANCR promoter between affected and unaffected tissues would help in identification of the impact of epigenetics on its expression.

**FIGURE 6:**
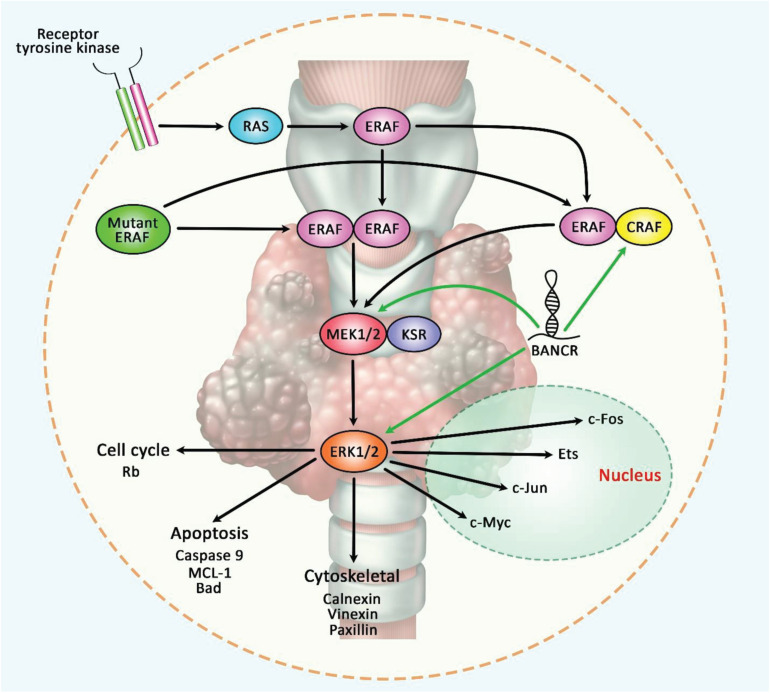
A schematic illustration of the oncogenic role of BANCR in modulating the RAF/MEK/ERK signaling pathway in papillary thyroid carcinoma. The MAPK-signaling cascade is generally triggered via the activation of a receptor tyrosine kinase that could play an important role in activating RAS, which in turn, facilitates homo- or heterodimerization of wild-type BRAF. Activated BRAF could have a potential role in phosphorylating MEK (which is bound to KSR) that could phosphorylate ERK, and thereby leading to various cellular effects including proliferation and survival. Mutant BRAF could trigger dimerization and activation of MEK without Ras activation (Caronia et al., 2011). Growing evidence has demonstrated that lncRNA BANCR could play a crucial role in enhancing epithelial-mesenchymal transition in papillary thyroid carcinoma via up-regulating the expression levels of CRAF, MEK1/2, and ERK1/2 in tumor cells (Wang et al., 2018a,b). Green arrows indicate the up-regulation of target genes modulated via lncRNA BANCR.

Finally, BANCR can be included in lncRNA-based diagnostic panels for early detection of cancers and monitoring cancer dynamics considering the association between its serum levels and clinical/pathological features of malignancy. Such application in liquid biopsy outperforms the conventional invasive and non-invasive methods of cancer detection. Prognostic value of BANCR has also been appraised in some types of cancers. A previous meta-analysis of data about expression of BANCR in gastrointestinal cancers has indicated correlation between over-expression of BANCR and some malignant characteristics such as lymph node or remote metastasis, clinical stage and poor overall survival (Fan et al., 2017). Figure 6 represents the oncogenic role of BANCR in regulating the RAF/MEK/ERK signaling pathway in papillary thyroid carcinoma.

Although therapeutic targeting of BANCR is still in its infancy, several strategies such as Anti-sense oligonucleotides (ASOs), modulation of gene expression using CRISPR-Cas9 or small molecules and therapeutic manipulation of the promoter region are putative therapeutic strategies which have been used in different settings (Arun et al., 2018). Sequence-based nucleic acid therapeutic strategies have been promising methods which are growing at a fast speed. However, many questions particularly those related with the safety and efficiency issues remain to be solved before translation of this field of basic science to clinical application. Targeting BANCR in a tissue-specific manner is another important issue which can be achieved through application of certain vectors with affinity to target tissues. Receptor-targeted adeno-associated viral vectors have been constructed with specificity for a number of tumor-associated proteins such as Her2/neu, EpCAM, or CD4 (Münch et al., 2015). Similar approaches can be designed to provide tissue as well as target specificity in this field.

In brief, BANCR is an lncRNA with bidirectional effects in the pathogenesis of cancers. Possible explanations for such effects are diversity and tissue-specificity of its targets, the presence of certain mutations which modulate BANR effects, and other confounding parameters which should be unraveled in future investigations.

## Author Contributions

MT and SG-F wrote the draft and revised it. BH, AA, TA, and HH collected the data and designed the figure and tables. All authors approved the submitted version.

## Conflict of Interest

The authors declare that the research was conducted in the absence of any commercial or financial relationships that could be construed as a potential conflict of interest.

## Publisher’s Note

All claims expressed in this article are solely those of the authors and do not necessarily represent those of their affiliated organizations, or those of the publisher, the editors and the reviewers. Any product that may be evaluated in this article, or claim that may be made by its manufacturer, is not guaranteed or endorsed by the publisher.
