# Supplementary material for: Role of lncRNA BANCR in Human Cancers: An Updated Review
Source: Front Cell Dev Biol. 2021 Aug 2;9:689992. doi: 10.3389/fcell.2021.689992 (PMC8367322; doi:10.3389/fcell.2021.689992)
Supplement: Supplementary file 1 [file Table_1.docx]

Table supplementary 1

Abnormal transcript levels of BANCR in cancer cell lines and the functional consequences of this dysregulation.

| Cancer | Upstream pathways | Downstream pathways | Cell lines | Expression (tumor cell lines) | Function | Conclusion | Reference |
| --- | --- | --- | --- | --- | --- | --- | --- |
| Endometrial Cancer-type 1 | - | ERK/MAPK and MMP1,MMP2 | Ishikawa and HEC-1A | Upregulated | Δ BANCR: ↓ proliferation, migration and invasion | BANCR played important role in the progression of endometrial cancer-type1. | ([5](#_ENREF_5)) |
| Uterine leiomyoma | - | - | Human Uterine leiomyoma cultured cells | Upregulated | Deoxyelephantopin caused BANCR down-regulation. | BANCR functioned as an oncogene in uterine leiomyoma. Deoxyelephantopin had an anti-tumor effect by downregulated oncogenic genes. | ([6](#_ENREF_6)) |
| Bladder cancer | - | - | T24, SW780 and SV-HUC1 | Downregulated | ↑ BANCR: ↓ proliferation, migration and ↑ apoptosis | BANCR was identified as a tumor suppressor in bladder cancer. | ([14](#_ENREF_14)) |
| Clear cell renal cell carcinoma | - | - | A498,786-O and HK-2 | Downregulated | ↑ BANCR: ↓ proliferation, migration, invasion and ↑ apoptosis and cell cycle arrest | BANCR was identified as a tumor suppressor in clear cell renal cell carcinoma. | ([15](#_ENREF_15)) |
| Colorectal cancer | - | miR-203/ CSE1L | HCT116, LoVo and NCM460 | Upregulated | Δ BANCR: ↓ proliferation, migration, invasion, and ↑ cell apoptosis and chemotherapy sensitivity (Adriamycin) | BANCR had oncogenic role in colorectal cancer and might have the potential to become a therapeutic target. | ([18](#_ENREF_18)) |
|  | - | p21 | SW480, HCT116, RQO, HT-29 and HCoEpiC | Downregulated | ↑ BANCR: ↓ proliferation and ↑ apoptosis | BANCR represented a tumor suppressor role in colorectal cancer. | ([16](#_ENREF_16)) |
|  | - | MEK | Caco‑2, HCT 116, LoVo, SW480, SW620, HT‑29 and CCD 841 CoN | Upregulated | Δ BANCR: ↓ migration and EMT | BANCR had oncogenic role in colorectal cancer progression. | ([7](#_ENREF_7)) |
|  | Ets-1/histone H3 | - | LOVO and SW480 | Downregulated | Fentanyl treatment caused BANCR expression and induced ↓ colony formation, cell migration and invasion | Fentanyl represented anti-tumor result in colorectal cancer that might be through regulatory role on BANCR and Ets-1. | ([17](#_ENREF_17)) |
| Gastric cancer | - | miR-9/ NF-KB1 | BGC823, SGC7901 and MGC803 | Upregulated | Δ BANCR: ↓ proliferation and ↑ cell apoptosis | BANCR played oncogenic role in gastric cancer through interaction with miR-9/ NF-KB1. | ([8](#_ENREF_8)) |
|  | - | - | HGC27, AGS, SGC-7901, MGC803 and GES-1 | Upregulated | - | BANCR played oncogenic role in gastric cancer. | ([19](#_ENREF_19)) |
|  | - | ERK1/2 | AGS, HGC27, SGC7901, MKN45 and GES-1 | Upregulated | ↑ BANCR: ↑ cell viability and colony formation in cisplatin treated cells | BANCR represented oncogenic role in gastric cancer and increased resistance to cisplatin via ERK1/2 pathway. | ([20](#_ENREF_20)) |
| Hepatocellular carcinoma | - | MEK, ERK and JNK | Huh7 and L-02 | Upregulated | Δ BANCR: ↓ viability, migration, invasion and ↑ apoptosis | BANCR had oncogenic role in hepatocellular carcinoma and might be a promising therapeutic target. | ([9](#_ENREF_9)) |
|  | - | - | HuH-7, Hep3B, HepG2, H2-M and CL-48 | Upregulated | Δ BANCR: ↓ proliferation, migration, invasion and EMT and ↑ apoptosis | BANCR had oncogenic role in hepatocellular carcinoma and might related to cancer initiation and progression. | ([10](#_ENREF_10)) |
| Pancreatic cancer | - | miR-195-5p/ Wnt/b-Catenin | PANC-1, SW1990, HS766T, CFPAC-1 and HPNE | Upregulated | Δ BANCR: ↓ proliferation, migration and invasion | BANCR might promote tumorigenesis through miR-195-5p/ Wnt/b-Catenin pathway in pancreatic cancer. | ([11](#_ENREF_11)) |
| Breast cancer | - | - | MCF-7, MDA-MB-231, SKBR3, BT-20 and MCF-10A | Upregulated | Δ BANCR: ↓ proliferation, migration and invasion capacities, EMT and ↑ cell apoptosis | Oncogenic role of BANCR in breast cancer took part in tumor progression. | ([21](#_ENREF_21)) |
|  | - | - | MDA-MB-468, MCF7, HCC1569, BT549, MDA-MB-231 and MCF10A | Upregulated | Δ BANCR: ↓ proliferation, EMT and metastasis | BANCR took part oncogenic role in breast cancer and promoted tumor progression. | ([22](#_ENREF_22)) |
| Lung carcinoma | - | p38 MAPK | NCI-H1688, NCI-H446, A549, SPC-A1 and 16HBE | Downregulated | ↑ BANCR: ↓ proliferation and migration | Tumor suppressor role of BANCR in ling carcinoma might be promising therapeutic target. | ([23](#_ENREF_23)) |
| Non-small cell lung cancer | Histone deacetylation | - | A549, SPC-A1, NCI-H1975, NCI-H1299, NCI-H1650, SK-MES-1 and 16HBE | Downregulated | ↑ BANCR: ↓ viability, migration, invasion, EMT and ↑ apoptosis | BANCR had tumor suppressor role in NSCLC and represented EMT regulatory role. | ([24](#_ENREF_24)) |
|  | - | - | A549, SPC-A1, H1299, H1650, H1975, PC-9 and 16HBE | Downregulated | ↑ BANCR: ↓ viability, invasion and ↑ apoptosis | Tumor suppressor role of BANCR in NSCLC led to regulate cell viability and invasion. | ([25](#_ENREF_25)) |
| Esophageal squamous cell carcinoma | - | Wnt/β-Catenin | KYSE30, TE10 and Het-1A | Upregulated | Δ BANCR: ↓ migration and invasion ability | Oncogenic role of BANCR in ESCC led to regulate migration and invasion through Wnt/β-Catenin pathway. | ([26](#_ENREF_26)) |
|  | - | - | KYSE-30, KYSE-70, KYSE-140, KYSE-150, KYSE-450, KYSE-510, TE-10, TE-12 and Het-1A | Upregulated | __ | BANCR had oncogenic role in ESCC. | ([27](#_ENREF_27)) |
|  | - | miR-338-3p/ IGF1R/Raf/MEK/ERK | KYSE450, KYSE510 and HET-1A | Upregulated | Δ BANCR: ↓ cell proliferation, migration, invasion and EMT | BANCR represented oncogenic role in ESCC progression through miR-338-3p/ IGF1R/Raf/MEK/ERK pathway. | ([28](#_ENREF_28)) |
|  | - | Raf/MEK/ERK | Eca-109, KYSE30, KYSE150, TE-1 and Het-1A | Upregulated | ↑ BANCR: ↑ proliferation, migration and invasion | BANCR played oncogenic role in ESCC through involvement of Raf/MEK/ERK pathway regulation. | ([29](#_ENREF_29)) |
| Papillary thyroid carcinoma | - | TSHR/cyclin D1, EZH2 | IHH-4 | Upregulated | Δ BANCR: ↓ TSHR expression and cell proliferation | BANCR act as an oncogene in PTC.it effected on TSHR expression through regulate chromatin recruitment of EZH2. | ([30](#_ENREF_30)) |
|  | - | Autophagy | IHH-4 | Upregulated | Δ BANCR: ↓ proliferation, autophagy and ↑ apoptosis | BANCR played oncogenic role in PTC through increased proliferation and activated autophagy. | ([31](#_ENREF_31)) |
|  | - | TSHR/cyclin D1 | IHH-4 | Upregulated | Δ BANCR: ↓ TSHR expression and cell proliferation | BANCR had an oncogenic role in PTC development.it might be through regulating TSHR and cyclin D1. | ([32](#_ENREF_32)) |
|  | - | MAPK and PI3K-Akt pathways | NPA, BCPAP, TPC1 and Nthy-ori3-1 | Downregulated | Δ BANCR: ↑ proliferation, invasion and ↓ apoptosis | BANCR functioned as a tumor suppressor in PTC and effected on MAPK and PI3K-Akt pathways. | ([33](#_ENREF_33)) |
|  | - | RAF/MEK/ERK | CAL-62, WRO, FTC-133, BCPAP and Nthy-ori 3-1 | Upregulated | ↑ BANCR: ↑cancer stem cell markers expression (LGR5 and EPCAM) | Oncogenic role of BANCR in PTC represented by regulating LGR5 and EPCAM through RAF/MEK/ERK pathway. | ([34](#_ENREF_34)) |
|  | - | Raf/MEK/ERK | BCPAP, CAL-62, WRO and FTC-133 | Upregulated | ↑ BANCR: ↑migration, invasion and EMT | Oncogenic role of BANCR in PTC led to EMT induction through Raf/MEK/ERK pathway. | ([35](#_ENREF_35)) |
|  | - | ERK1/2 and P38 | TPC-1, K1, BCPAP, CGTH-W3 and Nthy-ori 3-1 | Downregulated | ↑ BANCR: ↓ proliferation, migration, invasion and ↑ apoptosis | BANCR act as a tumor suppressor in PTC through ERK1/2 and P38 activation. | ([36](#_ENREF_36)) |
|  | - | TSHR/CCND1 | IHH-4, FTC133, and 8505C | Upregulated | Luteolin treatment caused BANCR downregulation and had anti-tumor effect | BANCR/TSHR oncogenic signaling inhibited anti-tumor effect of luteolin. | ([37](#_ENREF_37)) |
| Thyroid carcinoma | - | - | thyroid cancer cells | Upregulated | Δ BANCR: ↓ proliferation, invasion and ↑ apoptosis and autophagy | BANCR had an oncogenic role in thyroid carcinoma through influence on autophagy process. | ([38](#_ENREF_38)) |
| Oral squamous cell carcinoma | - | MAPK | CAL-27, SCC-4, SCC-9, SCC-25 and HOK | Upregulated | Δ BANCR: ↓ proliferation, migration, invasion and ↑ apoptosis | BANCR played in OSCC tumorigenesis in oncogenic manner via MAPK interaction. | ([39](#_ENREF_39)) |
| Retinoblastoma | - | miR-204-3p/Wnt/β-catenin | HXO-RB44, Y79, WERI-Rb1 and ARPE-19 | Upregulated | Δ BANCR: ↓ proliferation, migration and invasion and ↑ apoptosis | BANCR had an oncogenic role in retinoblastoma and affect tumorigenesis through Wnt/β-catenin signaling. | ([40](#_ENREF_40)) |
|  | - | - | Weri-Rb1, Y79, ARPE-19 and HRMECs | Upregulated | Δ BANCR: ↓ proliferation, migration and invasion | BANCR played oncogenic role in retinoblastoma and affect cellular aggressiveness. | ([41](#_ENREF_41)) |
| Melanoma | - | miR‑204/ Notch2 | A375, A875, M14 and human epidermal melanocytes | Upregulated | Δ BANCR: ↓ proliferation, migration and invasion | BANCR had oncogenic role in melanoma and through interaction with miR‑204/ Notch2 regulate tumor progression. | ([42](#_ENREF_42)) |
|  | - | ERK1/2 or JNK/MAPK | A-375, 1205Lu, UACC903, CHL-1 and sk-mel-5 | Upregulated | Δ BANCR: ↓ proliferation | BANCR oncogenic role in melanoma regulated proliferation through MAPK pathway. | ([43](#_ENREF_43)) |
|  | - | LINC-PINT | A375-P and A375-MA2 | Upregulated | Δ BANCR: ↓ proliferation | BANCR affect tumorigenesis through regulation of LINC-PINT. | ([44](#_ENREF_44)) |
|  | BRAFV600E | - | human melanocytes, 293T and sk-mel-5 | Upregulated | BRAF V600E induces BANCR expression and regulates cell migration. | BANCR had an oncogenic role in melanoma with potential function in migration. | ([2](#_ENREF_2)) |
| Osteosarcoma | - | Wnt signaling | MG-63 | Downregulated | Baicalein caused BANCR over expression that induced apoptosis. | Baicalein inhibited osteosarcoma cell proliferation through Wnt signaling that BANCR involved in the pathway. | ([45](#_ENREF_45)) |
