# Supplementary material for: Role of lncRNA BANCR in Human Cancers: An Updated Review
Source: Front Cell Dev Biol. 2021 Aug 2;9:689992. doi: 10.3389/fcell.2021.689992 (PMC8367322; doi:10.3389/fcell.2021.689992)
Supplement: Supplementary file 2 [file Table_2.docx]

Table supplementary 1

The observed effects of BANCR dysregulation in clinical settings (OS: overall survival, DFS: disease-free survival, RFS: relapse-free survival, ANCTs: adjacent non-cancerous tissues).

| Cancer types | Samples | Expression (tumor vs. normal) | Kaplan-Meier analysis | Univariate cox regression | Multivariate cox regression | Conclusion | Reference |
| --- | --- | --- | --- | --- | --- | --- | --- |
| Endometrial Cancer-type 1 | 30 cancer tissues and 20 myoma samples | Upregulated | - | - | - | BANCR played oncogenic role and its expression was correlated with patients clinicopathological variables (FIGO stage, pathological grade, myometrial invasion and lymph node metastasis). | ([5](#_ENREF_5)) |
| Epithelial ovarian cancer | 84 cancer tissues and 36 normal ovarian epithelial tissues | Upregulated | - | - | BANCR overexpression was an independent unfavorable prognostic factor. | BANCR functioned as an oncogene in epithelial ovarian cancer. Its expression was associated with FIGO stage, CA125 expression level and lymph node metastasis. | ([53](#_ENREF_53)) |
| Bladder cancer | 54 pairs of cancer tissues and matched-ANCTs | Downregulated | - | - | - | BANCR took part in bladder cancer as a tumor suppressor and its expression was correlated with TNM stage. | ([14](#_ENREF_14)) |
| Clear cell renal cell carcinoma | 62 pairs of cancer tissues and matched ANCTs | Downregulated | Higher BANCR expression was significantly associated with better prognosis. | - | - | BANCR played tumor suppressor role in clear cell renal cell carcinoma and was correlated with disease prognosis. | ([15](#_ENREF_15)) |
| Colorectal cancer | 32 pairs of cancer tissues and ANCTs | Upregulated | - | - | - | BANCR represented oncogenic role in colorectal cancer progression. | ([18](#_ENREF_18)) |
|  | 38 pairs of cancer tissues and ANCTs | Downregulated | - | - | - | BANCR had tumor suppressor role in colorectal cancer and its expression was correlated with pathological stage and larger tumor size. | ([16](#_ENREF_16)) |
|  | 60 pairs of CRC tissues and ANCTs | Upregulated | - | - | - | BANCR played oncogenic role in colorectal cancer and its expression was correlated with lymph node metastasis and tumor stage. | ([7](#_ENREF_7)) |
|  | 106 cancer tissues and 65 ANCTs | Upregulated | Higher BANCR expression was significantly correlated with lower OS. | Tumor size, Depth of invasion, Lymph node metastasis and BANCR expression were significant prognostic factors. | Tumor size, Lymph node metastasis and BANCR expression were independent prognostic factors. | BANCR had an oncogenic role in colorectal cancer. its expression was correlated with lymph node metastasis and disease prognosis. | ([54](#_ENREF_54)) |
|  | 80 pairs of cancer tissues and NM tissues  240 serum samples of patients and healthy controls | Upregulated | Higher BANCR serum expression level was significantly correlated with lower OS. |  |  | BANCR functioned in oncogenic manner in colorectal cancer. Its expression was associated with TNM stage, lymph node metastasis and local invasion. | ([55](#_ENREF_55)) |
| Gastric cancer | 30 pairs of cancer tissues and ANCTs | Upregulated | - | - | - | BANCR played oncogenic role in gastric cancer progression. | ([8](#_ENREF_8)) |
|  | 35 pairs of cancer tissues and ANCTs | Upregulated | - | - | - | BANCR played oncogenic role in gastric cancer. Its expression was associated with TNM stage and lymph node metastasis. | ([20](#_ENREF_20)) |
|  | 184 cancer tissues and 20 normal gastric tissues | Upregulated | Lower BANCR expression was correlated with better OS. | Clinical stage, tumor depth, lymph node metastasis, distant metastasis and BANCR expression were significant prognostic factors. | Clinical stage, tumor depth, distant metastasis and high BANCR expression levels were independent poor prognostic factors. | BANCR had an oncogenic role in gastric cancer. Its expression was correlated with clinicopathological characteristics and prognosis. | ([56](#_ENREF_56)) |
|  | 20 pairs of cancer tissues and ANCTs/167 GC plasma samples and 110 healthy samples | Upregulated | - | - | - | BANCR expression correlated with tumor size, depth of invasion, lymphatic metastasis and more advanced tumor stages. BANCR could be effective in diagnosis of gastric cancer patients from precancerous ones and individual with gastrointestinal stromal tumor. | ([19](#_ENREF_19)) |
| Hepatocellular carcinoma | 46 pairs of cancer tissues and ANCTs of HBV associated cancers | Downregulated | - | - | - | BANCR played tumor suppressor role in hepatocellular carcinoma and its expression was correlated with AFP levels and tumor numbers. | ([50](#_ENREF_50)) |
|  | 109 pairs of cancer tissues and ANCTs | Upregulated | Higher BANCR expression was significantly correlated with poor OS. | Tumor grade, tumor diameter, Venous infiltration, TNM stage and BANCR expression were significant prognostic factors. | Tumor diameter, presence of venous infiltration, TNM stage I-II and low BANCR expression were independent prognostic factors. | BANCR had an oncogenic role in hepatocellular carcinoma and its expression was correlated with clinicopathologic characteristics.it might be took part in initiation and progression of cancer. | ([10](#_ENREF_10)) |
| Pancreatic cancer | 45 pairs of cancer tissues and ANCTs | Upregulated | - | - | - | BANCR played oncogenic role and promote tumorigenesis of pancreatic cancer. | ([11](#_ENREF_11)) |
| Breast cancer | 65 pairs of cancer tissues and matched ANCTs | Upregulated | Higher BANCR expression was correlated with shorter OS and DFS. | - | - | Oncogenic role of BANCR in breast cancer was associated with tumor size, TNM stage, lymph node metastasis and survival. | ([21](#_ENREF_21)) |
|  | 216 pairs of cancer tissues and ANCTs | Upregulated | Higher BANCR expression was associated with poorer OS and reduced RFS. | Positive lymph node status, advanced TNM stage and high BANCR expression were significant risk factors for OS and RFS. | High BANCR expression level was independent poor prognostic factor for OS and RFS. | BANCR played oncogenic role in breast cancer and its expression level was correlated with clinicopathological characteristics and survival. | ([22](#_ENREF_22)) |
| Lung carcinoma | 12 pairs of cancer tissues and ANCTs | Downregulated | - | - | - | BANCR took part as a tumor suppressor in oncogenesis process of lung carcinoma. | ([23](#_ENREF_23)) |
| Non-small cell lung cancer | 113 pairs of cancer tissues and ANCTs | Downregulated | Lower BANCR expression was associated with shorter survival rate. | lymph node metastasis, TNM stage and BANCR expression level were significant prognostic factors. | low BANCR expression level was independent indicator of poor survival for patients. | BANCR played tumor suppressor role in NSCLC. its expression was correlated with TNM stage, tumor size, lymph node metastasis and survival. | ([24](#_ENREF_24)) |
|  | 27 pairs of cancer tissues and ANCTs | Downregulated | - | - | - | BANCR played tumor suppressor role in NSCLC. | ([25](#_ENREF_25)) |
|  | TCGA-LUAD data: 535 cancer samples and 59 non-tumor tissues  TCGA-LUSC data:  02 cancer samples and 49 non-tumor tissues | Downregulated | Lower BANCR expression was associated with better survival. | - | - | BANCR was one of the key lncRNA in NSCLC progression and its lower expression was associated with better survival rate. | ([57](#_ENREF_57)) |
| Esophageal squamous cell carcinoma | 80 pairs of cancer tissues and ANCTs | Upregulated | Higher BANCR expression was correlated with shorter OS. | - | - | BANCR oncogenic role in ESCC correlated with WHO grade, lymph node metastasis and survival. | ([26](#_ENREF_26)) |
|  | 40 pairs of cancer tissues and ANCTs | Upregulated | Higher BANCR expression was associated with shorter OS. | - | - | BANCR had an oncogenic role in ESCC. | ([28](#_ENREF_28)) |
|  | 142 pairs of cancer tissues and ANCTs/30 plasma samples before - after surgery and 30 controls | Upregulated | Higher BANCR expression was correlated with reduced OS and DFS. | Depth of invasion, lymph node metastasis, distant metastasis and BANCR expression were significant prognostic factors. | High BANCR expression level was independent poor prognostic factor. | BANCR oncogenic role in ESCC was correlated with WHO grade, lymph node metastasis, distant metastasis, clinical stage and survival. | ([27](#_ENREF_27)) |
|  | 75 pairs of cancer tissues and ANCTs | Upregulated | - | - | - | BANCR oncogenic role in ESCC was correlated with tumor differentiation, tumor stage, lymph node metastasis and 5 years survival. | ([58](#_ENREF_58)) |
| Papillary thyroid carcinoma | 40 pairs of cancer tissues and ANCTs | Upregulated | - | - | - | BANCR played oncogenic role in PTC. | ([30](#_ENREF_30)) |
|  | 6 pairs of cancer tissues and ANCTs | Upregulated | - | - | - | BANCR played oncogenic role in PTC. | ([31](#_ENREF_31)) |
|  | 32 malignant cancer tissues and 28 benign thyroid nodule samples as a control | Upregulated | - | - | - | BANCR functioned as an oncogene in PTC development. | ([32](#_ENREF_32)) |
|  | 60 pairs of cancer tissues and ANCTs | Downregulated | - | - | - | BANCR took part as a tumor suppressor in PTC and its expression was associated with lymph node metastasis. | ([33](#_ENREF_33)) |
|  | 30 pairs of cancer tissues and ANCTs | Upregulated | - | - | - | BANCR had an oncogenic role in PTC development. | ([34](#_ENREF_34)) |
|  | 27 pairs of cancer tissues and ANCTs | Upregulated | - | - | - | BANCR played oncogenic role in PTC. | ([35](#_ENREF_35)) |
|  | 92 pairs of cancer tissues and ANCTs | Downregulated | - | - | - | BANCR played as a tumor suppressor in PTC. Its expression was associated with tumor size, multifocal lesion and TNM stage. | ([36](#_ENREF_36)) |
|  | 85 pairs of cancer tissues and ANCTs | Downregulated (in BRAFV600E positive) | - | - | - | BANCR expression in presence of BRAFV600E mutation was associated with lymph node metastasis while in absence of this mutation; it was associated with thyroid capsule invasion. | ([51](#_ENREF_51)) |
|  | 51 pairs of cancer tissues and ANCTs | Upregulated | - | - | - | BRAFV600E mutation was correlated with BANCR up regulation and it was associated with higher extracapsular invasion and lateral LNM. | ([52](#_ENREF_52)) |
| Thyroid carcinoma | thyroid carcinoma and normal thyroid tissues | Upregulated | - | - | - | BANCR functioned as an oncogene in thyroid carcinoma. Its expression was associated with pathological stage of and lymph node metastasis. | ([38](#_ENREF_38)) |
| Oral squamous cell carcinoma | 50 pairs of cancer tissues and ANCTs | Upregulated | Higher BANCR expression was associated with shorter OS and DFS. | - | High BANCR expression level was independent factor for predicting poor OS and DFS. | BANCR played oncogenic role in OSCC and its expression was associated with Lymph node metastasis and survival. | ([39](#_ENREF_39)) |
| Retinoblastoma | 60 cancer tissues and 4 normal retina tissues | Upregulated | Higher BANCR expression was correlated with poor prognosis and shorter OS. | Tumor size, Choroidal invasion, Optic nerve invasion and BANCR expression were significant prognostic factors. | Negative Optic nerve invasion and lower BANCR expression were independent factors for predicting poor OS. | BANCR took part in retinoblastoma in oncogenic manner. Its expression associated with tumor size, choroidal invasion, optic nerve invasion and prognosis. | ([41](#_ENREF_41)) |
| Melanoma | 69 melanoma tissues and age/gender-matched controls | Upregulated | - | - | ­- | BANCR had an oncogenic role in melanoma. | ([42](#_ENREF_42)) |
|  | 103 melanoma tissues and 12 age/gender-matched control tissues | Upregulated | Higher BANCR expression was associated with shorter OS. | - | - | Oncogenic role of BANCR in melanoma was associated with TNM classification and survival. | ([43](#_ENREF_43)) |
|  | 60 pairs of melanoma tissues and ANCTs | Upregulated | - | - | - | BANCR played oncogenic role in melanoma through LINC-PINT regulation. | ([44](#_ENREF_44)) |
